# Cough and urticarial rash in an 11-year-old child

**DOI:** 10.1590/0037-8682-0162-2023

**Published:** 2023-07-24

**Authors:** Maria Francesca Gicchino, Alma Nunzia Olivieri

**Affiliations:** 1 University of the Study of Campania “Luigi Vanvitelli”, Department of Woman, Child and General and Specialized Surgery, Naples, Italy. University of the Study of Campania “Luigi Vanvitelli” Department of Woman, Child and General and Specialized Surgery Naples Italy

**FIGURE 1:**
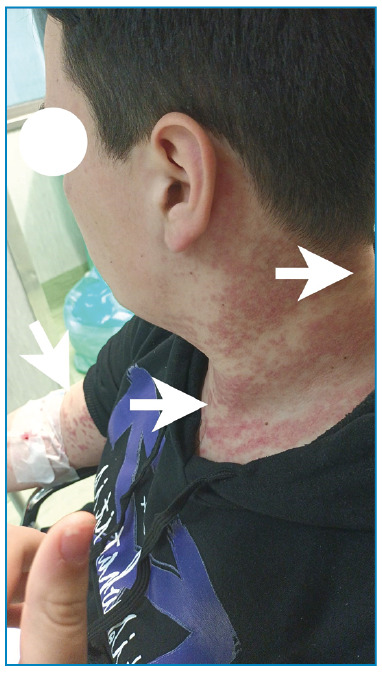
Urticarial rashes, showed by arrows

A 10-year-old boy was admitted to our department with urticarial rashes on the face, trunk, and upper and lower limbs. After allergological evaluation, the patient was administered with antihistamines (cetirizine 10 mg twice); however, the condition did not improve. He visited our department with a persistent urticarial rash and lower limb pain. Clinical evaluation revealed urticarial rashes on the face, trunk, and upper and lower limbs (Figure 1). Chest auscultation revealed left basal reduction of the vesicular murmur and diffuse expiratory rumors. Joint examinations did not reveal arthritis or enthesitis. 

**FIGURE 2:**
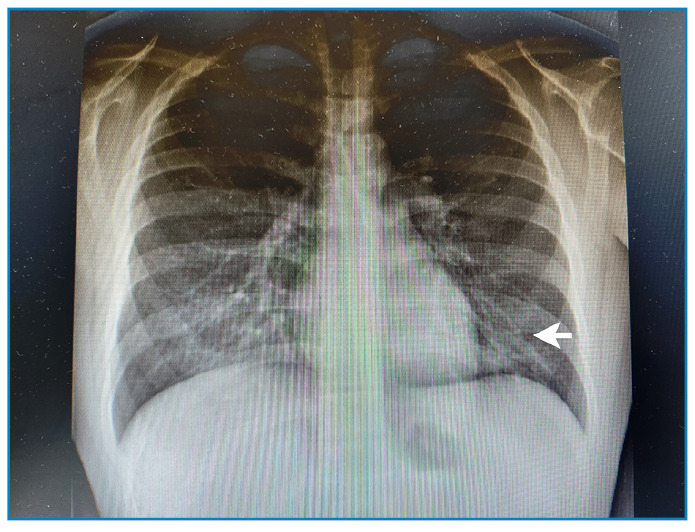
Chest radiograph: Opacity in the perihilar left field showed by the arrow.

Chest radiography revealed an opacity in the perihilar left field (Figure 2).

Blood examination revealed elevated C-reactive protein levels (1.5 mg/dL; normal value < 1 mg/dL).

Based on the chest auscultation findings, we evaluated the *M. pneumoniae* serology and found more than a four-fold increase in the titer of IgM and IgG antibodies (43 and 63 biological units/mL, respectively; normal value < 10 Bu/mL). Therefore, we administered clarithromycin (500 mg twice for 20 days), prednisone (2 mg/daily tapered in 2 weeks), and bronchodilators (salbutamol, four puffs four times per day, tapered in 1 week)^1^, which improved the clinical condition, and the urticarial rashes disappeared. In up to 30% of patients with *M. pneumoniae* infection, skin manifestations are evident; the most common are: exanthematous skin eruptions (8-33%), erythema nodosum (8%), and urticaria (7%)^2^. 

In patients with persistent urticaria, *M. pneumoniae* infection should be considered to make a correct diagnosis and administer adequate treatment^3^. 
